# Nutlin-3a suppresses poly (ADP-ribose) polymerase 1 by mechanisms different from conventional PARP1 suppressors in a human breast cancer cell line

**DOI:** 10.18632/oncotarget.27581

**Published:** 2020-05-05

**Authors:** Masaki Kobayashi, Yuka Ishizaki, Mika Owaki, Yoko Matsumoto, Yuri Kakiyama, Shunsuke Hoshino, Ryoma Tagawa, Yuka Sudo, Naoyuki Okita, Kazunori Akimoto, Yoshikazu Higami

**Affiliations:** ^1^Laboratory of Molecular Pathology & Metabolic Disease, Faculty of Pharmaceutical Sciences, Tokyo University of Science, Noda, Chiba 278-8510, Japan; ^2^Translational Research Center, Research Institute of Science and Technology, Tokyo University of Science, Noda, Chiba 278-8510, Japan; ^3^Division of Pathological Biochemistry, Faculty of Pharmaceutical Sciences, Sanyo-Onoda City University, Sanyo-onoda, Yamaguchi 756-0884, Japan; ^4^Laboratory of Medicinal and Life Science, Faculty of Pharmaceutical Sciences, Tokyo University of Science, Noda, Chiba 278-8510, Japan; ^*^Co-first authors

**Keywords:** nutlin-3a, PARP1, breast cancer, proteasomal degradation, autoPARylation

## Abstract

Poly (ADP-ribose) polymerase 1 (PARP1) plays important roles in single strand DNA repair. PARP1 inhibitors enhance the effects of DNA damaging drugs in homologous recombination-deficient tumors including tumors with breast cancer susceptibility gene (BRCA1) mutation. Nutlin-3a, an analog of cis-imidazoline, inhibits degradation of murine double minute 2 (MDM2) and stabilizes p53. We previously reported that nutlin-3a induces PARP1 degradation in p53-dependent manner in mouse fibroblasts, suggesting nutlin-3a may be a PARP1 suppressor. Here, we investigated the effects of nutlin-3a on PARP1 in MCF-7, a human breast cancer cell line. Consistent with our previous results, nutlin-3a reduced PARP1 levels in dose- and time-dependent manners in MCF-7 cells, but this reduction was suppressed in p53 knockdown cells. RITA, a p53 stabilizer that binds to p53 itself, failed to reduce PARP1 protein levels. Moreover, transient MDM2 knockdown repressed nutlin-3a-mediated PARP1 reduction. The MG132 proteasome inhibitor, and knockdown of checkpoint with forkhead and ring finger domains (CHFR) and ring finger protein 146 (RNF146), E3 ubiquitin ligases targeting PARP1, suppressed nutlin-3a-induced PARP1 reduction. Short-term nutlin-3a treatment elevated the levels of PARylated PARP1, suggesting nutlin-3a promoted PARylation of PARP1, thereby inducing its proteasomal degradation. Furthermore, nutlin-3a-induced PARP1 degradation enhanced DNA-damaging effects of cisplatin in BRCA1 knockdown cells. Our study revealed that nutlin-3a is a PARP1 suppressor that induces PARP1 proteasomal degradation by binding to MDM2 and promoting autoPARylation of PARP1. Further analysis of the mechanisms in nutlin-3a-induced PARP1 degradation may lead to the development of novel PARP1 suppressors applicable for cancers with BRCA1 mutation.

## INTRODUCTION

The poly (ADP-ribose) polymerase (PARP) family consists of 17 enzymes and functions in several cellular processes including DNA recombination and repair, cellular proliferation, apoptosis in ischemic conditions and necrotic cell death [1–3]. PARPs catalyze poly (ADP-ribosyl) ation (PARylation) of target proteins with intracellular nicotinamide adenine dinucleotide (NAD^+^). PARP1 is activated by DNA damage caused by free radicals or other agents and plays an important role in single strand DNA repair through the base excision pathway [1–3]. Activated PARP1 PARylates and recruits DNA repair factors such as X-ray repair cross-complementing gene 1 (XRCC1), DNA ligase III and polynucleotide kinase (PNK) to damaged sites, resulting in DNA repair [4]. PARP1 is also involved in transcriptional regulation and maintenance of genomic integrity via inducing chromatin structural change by PARylation of histones and DNA demethylase [5].

Previous studies have shown that PARylation enhances the ubiquitination of substrate proteins and thus promotes proteasomal degradation of several target proteins. Some E3 ubiquitin ligases have been reported to recognize the PAR-group on target proteins via the WWE or PBZ domain, resulting in increased target protein ubiquitination [6, 7]. Furthermore, PARP1 is regulated by ubiquitination-mediated proteosomal degradation, and ubiquitination of PARP1 has been shown to be dependent on PARylation by PARP1 itself, in other words via autoPARylation [8, 9]. For example, ring finger protein 146 (RNF146) induces poly-ubiquitination and degradation of autoPARylated PARP1, regulating the DNA damage response [10]. Likewise, checkpoint with forkhead and ring finger domains (CHFR) preferentially interacts with autoPARylated PARP1 under mitotic stress conditions and then induces PARP1 degradation via the ubiquitin-proteasome system [11]. These findings suggest that autoPARylation is important for the degradation of PARP1 protein.

Targeting the PARP1-mediated DNA repair pathway has recently received considerable attention as an approach for enhancing the effects of anti-cancer drugs and radiation therapy [12]. PARP1 inhibitors have been proven to enhance the sensitivity of homologous recombination (HR)-deficient tumors to DNA-damaging agents [13, 14]. PARP1 inhibitors are expected to serve as therapeutic agents for various cancers such as breast cancer susceptibility gene 1 (BRCA1)- and BRCA2-associated hereditary breast and ovarian cancers, prostate cancer, non-small cell lung cancer, and Ewing sarcoma [13–18]. For example, olaparib, the first available and approved PARP1/2 inhibitor, significantly extended the progression-free survival of BRCA1/2 mutation-positive ovarian cancer patients in clinical trials [19]. Moreover, olaparib monotherapy has provided a significant benefit over standard therapy for patients with HER2-negative metastatic breast cancer and a germline *BRCA* mutation [20]. Several studies have described mechanisms of actions of PARP inhibitors other than via DNA repair pathways, including metastasis, tumor angiogenesis and neuronal death [18, 21, 22]. Some available PARP1 inhibitors, many of which contain a nicotinamide/benzamide pharmacophore group, competitively inhibit the binding of PARP1 to NAD^+^ [23, 24].

Nutlin-3a, an analog of cis-imidazoline, potently binds the p53-binding domain in murine double minute 2 (MDM2), an E3 ubiquitin ligase for p53 tumor suppressor. Nutlin-3a interrupts the interaction between MDM2 and p53 and stabilizes p53 [25]. These cis-imidazoline analogs exhibit an inhibitory effect on the growth of various cancer cell lines and are in early phase clinical trials [26]. We previously reported that nutlin-3a induces proteasome-dependent PARP1 protein degradation in p53-dependent manner in mouse fibroblasts and increases p53 protein levels [27]. These discoveries provide the possibility of nutlin-3a as a PARP1 suppressor with a novel molecular mechanism. In the present study, we investigated this possibility by exploring the mechanisms of PARP1 reduction by nutlin-3a using the MCF-7 human breast cancer cell line.

## RESULTS

### Nutlin-3a downregulates PARP1 proteins levels in human breast cancer cells in a p53-dependent manner

**Figure 1:**
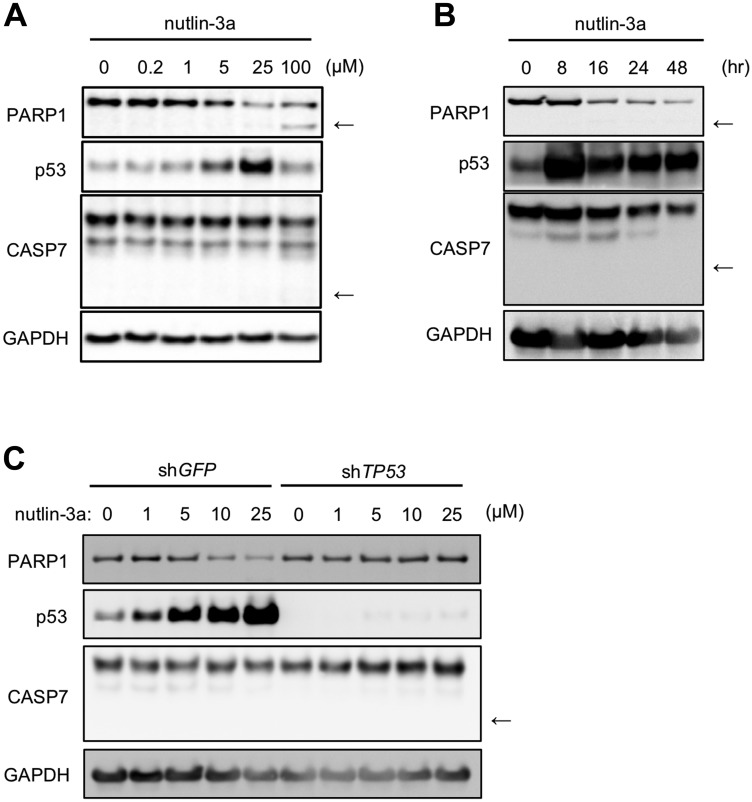
Nutlin-3a reduces PARP1 protein levels in MCF-7 cells, a human breast cancer cell line. (**A**) MCF-7 cells were treated with indicated concentrations of nutlin-3a for 24 h. (**B**) MCF-7 cells were treated with 10 μM nutlin-3a for the indicated times. (**C**) MCF-7/sh*GFP* and MCF-7/sh*TP53* cells were treated with indicated concentrations of nutlin-3a for 24 h. The cell lysates were analyzed by immunoblotting using the indicated antibodies. In the PARP1 and CASP7 panels, arrows indicate apoptotic fragments. GAPDH was used as a loading control.

In this study, we used the MCF-7 breast cancer cell line (p53 wild-type; estrogen receptor (+); progesterone receptor (+); Her2 (–)), which is widely used by many researchers. Treatment of MCF-7 cells with 5 μM and 25 μM nutlin-3a reduced PARP1 protein levels and increased p53 protein in a dose-dependent manner (Figure 1A). In contrast, 100 μM nutlin-3a induced cleavage of PARP1 and failed to increase p53 protein. Consistent with these results, MCF-7 cells treated with 100 μM nutlin-3a were detached from the culture dish, appearing to undergo cell death (data not shown). We did not detect cleaved Caspase 7 (CASP7) at any concentration of nutlin-3a (Figure 1A). We also found that nutlin-3a reduced PARP1 protein levels and exerted no influence on the cleavage of both PARP1 and CASP7 over 48 h in a time-dependent manner (Figure 1B).

We previously reported that nutlin-3a-induced reduction of PARP1 proteins occurs in a p53-dependent manner [27]. Hence, we generated MCF-7 cells expressing shRNA against TP53 to evaluate the p53-dependency in more detail. Down-regulation of p53 protein levels was confirmed in MCF-7/sh*TP53* cells (Figure 1C). Nutlin-3a treatment reduced PARP1 protein level in MCF-7 cells, but not in MCF-7/sh*TP53* cells (Figure 1C). Both cell lines exhibited no change in cleaved CASP7 levels after treatment of nutlin-3a. These results indicate that the nutlin-3a-induced reduction of PARP1 protein was dependent on p53 in human breast cancer cells but did not induce cell death, as observed in mouse fibroblasts.

### MDM2 is required for nutlin-3a-mediated reduction of PARP1 protein

**Figure 2:**
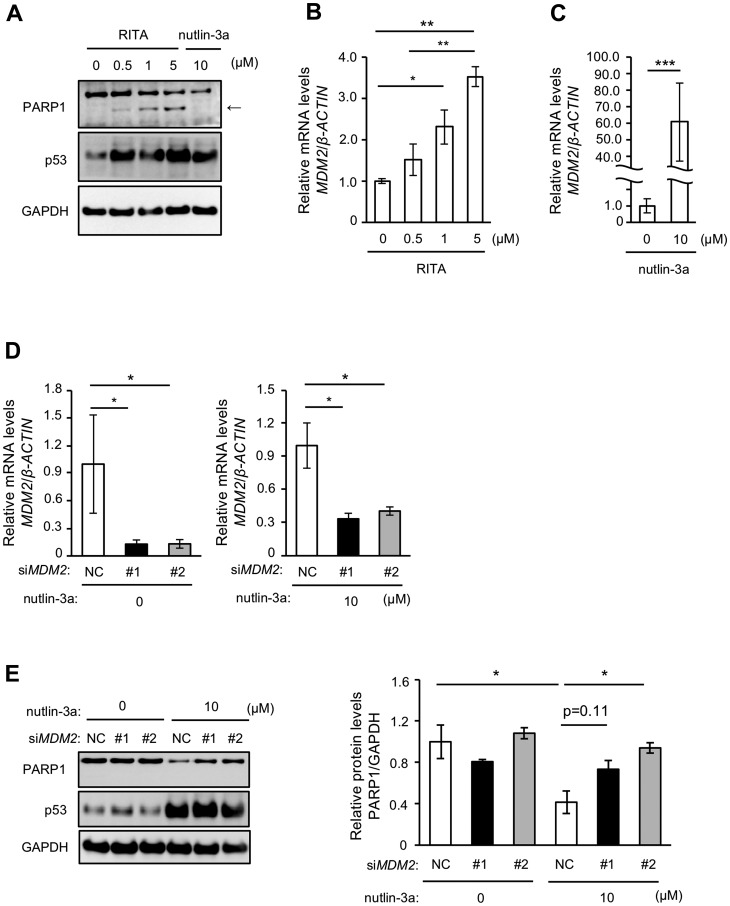
MDM2 is involved in nutlin-3a-induced PARP1 protein reduction. (**A**) MCF-7 cells were treated with indicated concentrations of RITA or 10 μM nutlin-3a for 24 h. The cell lysates were analyzed by immunoblotting using indicated antibodies. In the PARP1 panel, the arrow indicates apoptotic fragment of PARP1. (**B**, **C**) qRT-PCR was performed in MCF-7 cells treated with RITA (B) or nutlin-3a (C) at concentrations indicated in (A). (**D**) MCF-7 cells were transfected with two different sets of siRNA against *MDM2*. *MDM2* levels were analyzed by qRT-PCR. *β-ACTIN* was used as a housekeeping gene. (**E**) MCF-7 cells transfected with siRNAs were treated with 10 μM nutlin-3a for 24 h. The left panel shows a representative immunoblot of three different experiments. The right panel shows the quantitative data. NC indicates negative control. GAPDH was used as a loading control. Values in (B–E) are means ± SEM (*n* = 3; different experiments). Differences between values were analyzed by Student’s *t*-test for (C), Dunnett’s test for (D) or Tukey’s test for (B, E) (^*^ *p* < 0.05; ^**^ *p* < 0.01; ^***^ *p* < 0.005).

RITA has been shown to stabilize p53 by directly binding to p53 [28], unlike nutlin-3a, which stabilizes p53 by interacting with MDM2. We thus next examined the effect of RITA on PARP1 protein levels. RITA treatment stabilized p53 in MCF-7 cells, but interestingly increased levels of a cleaved form of PARP1 rather than reduced PARP1 levels (Figure 2A), suggesting that an increased amount of p53 itself is not critical for the reduction of PARP1 protein. In addition, RITA upregulated the mRNA level of *MDM2*, and this increase was more prominent after treatment with nutlin-3a (Figure 2B and 2C). We then performed transient knockdown (KD) of MDM2 in MCF-7 cells using siRNA (Figure 2D) and examined the effect of nutlin-3a-induced reduction of PARP1 protein. *MDM2* KD suppressed the nutlin-3a-induced decrease of PARP1 protein (Figure 2E). These results suggested that MDM2 is critical for nutlin-3a-induced PARP1 reduction.

### Nutlin-3a-induced PARP1 reduction is mediated by proteasomal degradation

**Figure 3:**
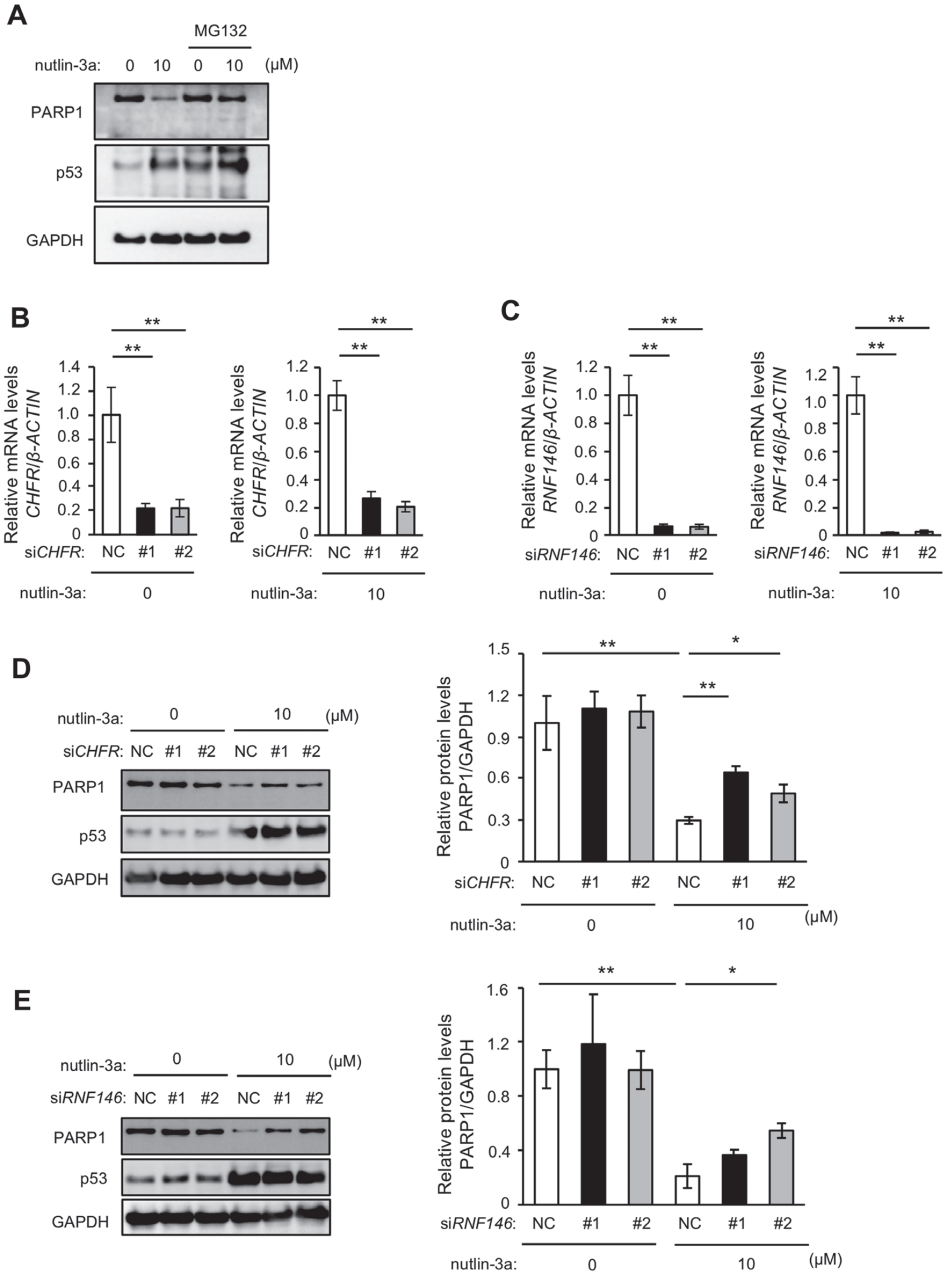
Nutlin-3a-induced PARP1 protein reduction is mediated by proteasomal degradation. (**A**) MCF-7 cells were treated with 10 μM nutlin-3a for 24 h. The proteasome inhibitor MG132 was directly added at 8 h after treatment with nutlin-3a. The cell lysates were analyzed by immunoblotting using indicated antibodies. (**B**, **C**) MCF-7 cells were transfected with two sets of siRNA against *CHFR* or *RNF146*. *CHFR* (B) and *RNF146* (C) levels were analyzed by qRT-PCR. *β*-*ACTIN* was used as a housekeeping gene. (**D**, **E**) MCF-7 cells transfected with siRNAs were treated with 10 μM nutlin-3a for 24 h. The left panels show representative immunoblotting images of three different experiments. The right panels show the quantitative data. GAPDH was used as a loading control. Values in (B–E) are means ± SEM (*n* = 3; different experiments). Differences between values were analyzed by Dunnett’s test for (B, C) or Tukey’s test for (D, E) (^*^ *p* < 0.05, ^**^ *p* < 0.01).

We previously demonstrated that nutlin-3a-induced PARP1 reduction in a mouse fibroblast cell line occurs via proteasome degradation [27]. To examine whether proteosomal degradation is involved in MCF-7 cells, we investigated nutlin-3a-induced PARP1 reduction after treatment with MG132, a proteasome inhibitor. Consistent with the mouse fibroblast results, MG132 treatment suppressed the reduction of PARP1 proteins by nutlin-3a (Figure 3A). Furthermore, nutlin-3a did not reduce mRNA levels of *PARP1* in MCF-7 cells (data not shown). E3 ubiquitin ligases play a central role in the ubiquitin-proteasome system by recognizing and ubiquitinating specific substrates. We thus speculated that nutlin-3a-induced PARP1 degradation may be mediated by E3 ubiquitin ligases for PARP1, such as CHFR and RNF146 [10, 11]. Transient KD of *CHFR* and *RNF146* in MCF7 cells was performed using siRNA (Figure 3B and 3C). Nutlin-3a-induced PARP1 degradation was suppressed in *CHFR* KD MCF7 cells (Figure 3D) and *RNF146* KD MCF7 cells (Figure 3E). These results suggested that nutlin-3a treatment promoted proteasome-mediated degradation of PARP1 proteins by CHFR and RNF146.

### AutoPARylation of PARP1 plays important roles in nutlin-3a-induced PARP1 degradation

**Figure 4:**
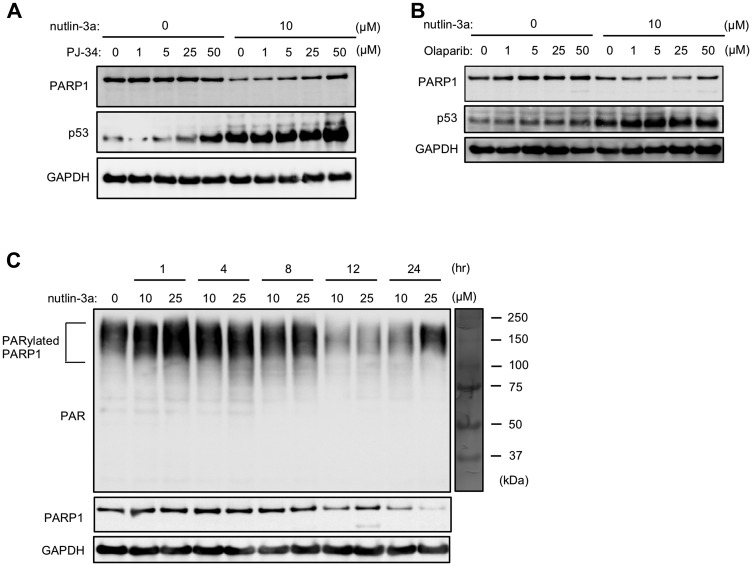
AutoPARylation of PARP1 plays important roles in nutlin-3a-induced PARP1 degradation. (**A**, **B**) MCF-7 cells were treated with 10 μM nutlin-3a in the presence or absence of the indicated concentrations of PJ34 (A) or olaparib (B) for 24 h. (**C**) MCF-7 cells were treated with 10 or 25 μM nutlin-3a for indicated times. Cell lysates were analyzed by immunoblotting using indicated antibodies. GAPDH was used as a loading control.

Several E3 ubiquitin ligases targeting PARP1 including CHFR and RNF146 recognize the PAR on PARylated PARP1 protein [10, 11]. Other studies reported that PARP1 has autoPARylation activity [8, 9]. Thus, we hypothesized that the nutlin-3a-induced PARP1 protein degradation may involve the targeting of E3 ubiquitin ligases to PARylated PARP1. We found that PJ34 and olaparib, which are PARP1/2 inhibitors that impair PARP1 activity via competitive inhibition of the binding of PARP1 to NAD^+^, suppressed nutlin-3a-induced degradation of PARP1 protein. These results implied that the action of nutlin-3a may be dependent on the PARylation activity of PARP protein itself (Figure 4A and 4B). In addition, to evaluate the effect of nutlin-3a on autoPARylation in nutlin-3a-induced PARP1 degradation, we examined changes in PARylated PARP1 levels in a time course assay. Several studies have shown that PARylated PARP1 bands are detected in molecular weights ranging from 100 kDa–200 kDa by immunoblotting of whole cell extracts with the anti-PAR antibody [29–32]. We were also able to detect PARylated PARP1 using immunoblotting and found that short-term treatment of nutlin-3a enhanced PARylated PARP1 levels prior to PARP1 degradation (Figure 4C). These results suggested that autoPARylation of PARP1 contributed to nutlin-3a-induced PARP1 degradation.

### Nutlin-3a enhances the effects of a DNA damaging agent in BRCA1 knockdown MCF-7 cells

**Figure 5:**
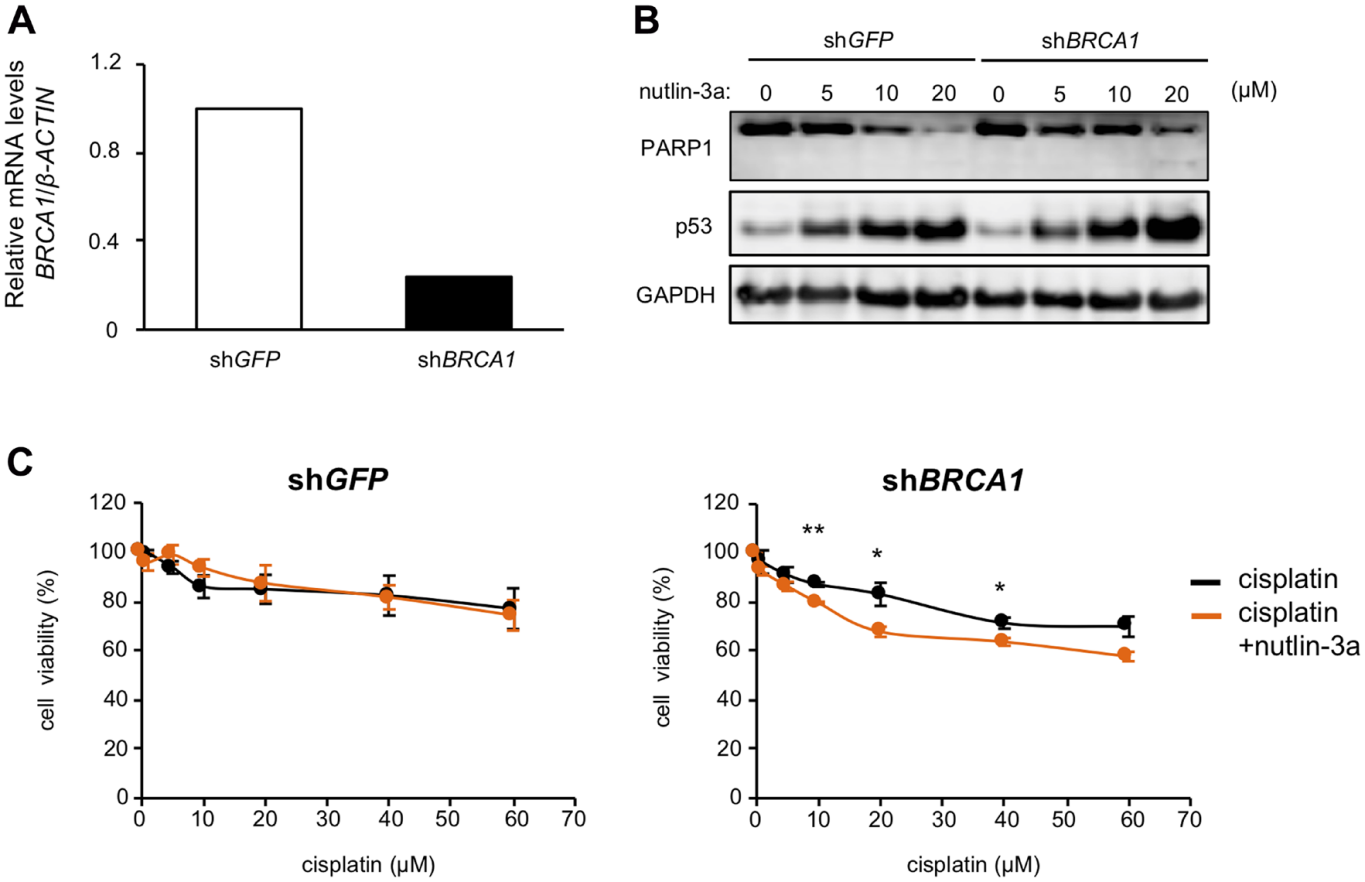
Nutlin-3a enhances the effect of cisplatin in *BRCA1* knockdown MCF-7 cells. (**A**) *BRCA1* levels in MCF-7/sh*GFP* and MCF-7/sh*BRCA1* cells were analyzed by qRT-PCR. (**B**) MCF-7/sh*GFP* and MCF-7/sh*BRCA1* cells were treated with nutlin-3a at the indicated concentrations for 24 h. Cell lysates were analyzed by immunoblotting using the indicated antibodies. (**C**) MCF-7/sh*GFP* (left panel) and MCF-7/sh*BRCA1* cells (right panel) were treated with 10 μM nutlin-3a. After 12 h, the indicated concentrations of cisplatin were directly added to pretreated cells and cells were incubated for 24 h. Cell viability was measured by WST-8 assay. Values are means ± SEM (*n* = 3; different experiments). Differences between values in each indicated concentration of cisplatin were analyzed by Student *t* test. (^*^ *p* < 0.05, ^**^ *p* < 0.01).

To evaluate whether nutlin-3a could serve as a PARP suppressor against BRCA1-associated hereditary breast cancer, we examined the effects of nutlin-3a in MCF-7 cells expressing shRNA against BRCA1 (MCF-7/sh*BRCA1*) or GFP (MCF-7/sh*GFP*), used as a negative control. In MCF-7/sh*BRCA1* cells with confirmed *BRCA1* knockdown, nutlin-3a-induced PARP1 degradation was partially inhibited (Figure 5A, 5B). We next compared cell viability between MCF-7/sh*BRCA1* and MCF-7/sh*GFP* cells treated with cisplatin, a DNA damaging agent, or cisplatin combined with nutlin-3a for 24 h after pretreatment with nutlin-3a for 12 h. The results showed that combination treatment of cisplatin and nutlin-3a decreased cell viability in MCF-7/sh*BRCA1* cells, but not in MCF-7/sh*GFP* cells (Figure 5C). These results suggested that nutlin-3a may enhance the effects of DNA damaging agents in BRCA1-mutated breast cancer cells.

## DISCUSSION

In the present study, we confirmed that nutlin-3a decreased PARP1 protein levels via the proteasome in a p53-dependent manner in MCF-7 cells, similar to its activity in mouse fibroblasts. We previously demonstrated that p53 overexpression has no effect on PARP1 protein levels, indicating that stabilized p53 is not crucial for nutlin-3a-induced degradation of PARP1 [27]. Our results with RITA were consistent with this previous report. Furthermore, both nutlin-3a and RITA upregulated *MDM2* levels in MCF-7 cells, probably from the transcriptional activation by accumulated p53, the extent of which was more prominent in cells treated with nutlin-3a. We found that knockdown of MDM2 also inhibited nutlin-3a-induced degradation of PARP1, underscoring the important role of MDM2 in the action of nutlin-3a on PARP1. This action mechanism is unclear at present, but we propose one possibility. Several previous reports have identified effects of nutlin-3a on MDM2 other than inhibiting p53 degradation. Wallace and colleagues found that in addition to MDM2 activities in the ubiquitin-proteasome system, MDM2 also activates target proteins through an allosteric mechanism [33]. The authors indicated that nutlin-3a can act as an allosteric agonist by affecting multiple MDM2 binding sites, as well as a p53 stabilizer [33]. Additionally, Nicholson and colleagues performed proteome analysis of MCF-7 cells treated with nutlin-3a and identified several novel proteins that interact with allosterically altered MDM2, such as nucleophosmin [34]. Notably, another report showed that nucleophosmin interacts with PARP1, although the contribution of nucleophosmin to PARP1 stability or activity is unclear [35]. Although the possibility that MDM2 might directly target PARP1 cannot be entirely ruled out, these studies suggest that nutlin-3a-induced PARP1 degradation might involve protein profile changes that result from the allosteric effects of nutlin-3a on MDM2. Based on these studies and our current data, we speculate that the binding of nutlin-3a to MDM2 can enhance the transcription of MDM2 via accumulated p53 and simultaneously induce allosteric alternations of increased MDM2, and this combination may cause reduced PARP1 protein levels.

We found that CHFR and RNF146 were involved in the effects of nutlin-3a using a knockdown approach. CHFR has a single PAR-binding zinc finger (PBZ), which is a PAR-binding motif [31]. RNF146 possesses the WWE domain, which contains four residues critical for binding to PAR [36]. These E3 ubiquitin ligases target PARP1 by recognizing and polyubiquitinating PARylated PARP1 [10, 11]. Our results of combination treatment of nutlin-3a with PJ34 or olaparib indicated that autoPARylation can contribute to nutlin-3a-induced PARP1 degradation. Of note, short-term treatment of nutlin-3a transiently enhanced PARylation of PARP1, whereas these levels were thereafter reduced in correlation with PARP1 protein levels. These data indicate that nutlin-3a might promote autoPARylation of PARP1 and subsequently induce PARP1 degradation by PARylation-associated targeting of E3 ubiquitin ligases. AutoPARylation of PARP1 is generally involved in various stress responses, including the DNA damage response and the heat shock response [37, 38]. Although the detailed mechanisms and regulation of autoPARylation of PARP1 currently remain to be explained, we propose the following putative mechanism. AutoPARylation of PARP1 is reportedly negatively regulated by PAR glycohydrolase (PARG), an enzyme responsible for the degradation of PAR [39]. In addition, a previous study demonstrated that PARG undergoes proteasomal degradation mediated by a RING-type E3 ubiquitin ligase [40]. Given that MDM2 is a RING-type E3 ubiquitin ligase, we hypothesize that nutlin-3a-induced allosteric changes of MDM2 could promote the autoPARylation of PARP1 possibly through the regulation of PARG.

PARP1 inhibitors disturb the single strand repair that causes persistent double strand breaks and lethality in BRCA1-deficient cancers lacking the capacity for HR repair [41–43]. However, several studies have described the limited efficacy of PARP1 inhibitors in some BRCA1 mutation carriers [44]. As a mechanism in such resistance, it has been demonstrated that ATR regulates BRCA1-independent HR by activating Rad51, which constitutes a complex with other Rad proteins and BRCA1 that is important for HR [45, 46]. Yazinski also revealed that ATR inhibition overcomes PARP1 inhibitor resistance in BRCA1-deficient cells [45]. Another group also reported Rad51-mediated resistance to PARP inhibition in triple negative breast cancers and breast cancer stem cells [47]. Furthermore, Ireno and colleagues demonstrated that nutlin-3a reduces Rad51 protein levels probably in a p53-dependent manner and suppresses homologous double strand break repair frequencies. This observation is supported by another previous report showing that p53 transcriptionally downregulated Rad51 [48, 49]. These findings indicate that nutlin-3a could be a PARP1 suppressor efficient for cancers with BRCA1 mutation and Rad51-dependent resistance to PARP1 inhibitors. Apart from the above, several reports have illustrated the tumor’s resistance to PARP1 competitive inhibitors, mimics of NAD^+^ such as olaparib and iniparib. For example, c-Met, a receptor tyrosine kinase that is overexpressed in various cancers, phosphorylates PARP1 at Tyr907 [50]. This phosphorylation causes reduced binding activity of PARP1 competitive inhibitors, which in turn develops the PARP1 inhibitor-resistance in cancer cells [50]. Additionally, another group highlighted that the loss of PARG, an enzyme responsible for degradation of PAR, is frequently observed in PARP1 inhibitor-resistant tumors [51]. PARG inactivation was also proven to cause accumulation of PAR, resulting in the disturbance of competitive inhibition of PARP1 activity [51]. Nutlin-3a may be able to avoid such PARP1 inhibitor-resistance, because nutlin-3a decreased PARP1 proteins, unlike the conventional inhibitors.

Nutlin-3a has been widely recognized as a p53 stabilizer. Our previous and present studies further demonstrate that nutlin-3a is a PARP1 suppressor with the ability to promote degradation of PARP1 protein. Moreover, we previously confirmed that Caylin2, a derivative of nutlin-3a, also induces PARP1 degradation [52]. This finding raises the possibility of nutlin-3a as a lead compound for the identification of more potent PARP1 suppressors with distinct mechanisms from the currently available PARP inhibitors. Further analysis of the effects of nutlin-3a will be important for the development of novel strategies of refractory cancer therapy.

## MATERIALS AND METHODS

### Cell culture and reagents

MCF-7 cells were obtained from the American Type Culture Collection (ATCC, Manassas, VA, USA) and cultured in Eagle’s Minimal Essential Medium (Wako, Osaka, Japan) with 10% fetal bovine serum (FBS) (Thermo Fisher Scientific, Waltham, MA, USA), 1% penicillin streptomycin (P/S) (Sigma-Aldrich, St. Louis, MO, USA), 10 μg/mL insulin (Wako), 1% MEM Non-essential Amino Acids Solution (Wako) and 1 mM Sodium Pyruvate Solution. Plat-A cells were kindly provided by Toshio Kitamura and cultured in DMEM (high glucose) with 10% FBS, 1% P/S, 1 μg/mL puromycin (Wako) and 10 μg/mL blasticidin (Funakoshi, Tokyo, Japan). Nutlin-3a was supplied by Cayman (Ann Arbor, MI, USA). The chemical structure of nutlin-3a has been shown in previous studies [25, 52]. RITA was purchased from Adooq Bioscience (Irvine, CA, USA). Olaparib was purchased from ChemScene, LLC (Monmouth Junction, NJ, USA). MG132, PJ34 and cisplatin were purchased from Wako. Cisplatin was dissolved in 90% dimethyl sulfoxide in phosphate buffered saline (90% DMSO in PBS) before use. Other reagents were dissolved in DMSO.

### Establishment of MCF-7 cells expressing shRNAs

For the p53 and BRCA1 stable KD cells, we designed human *TP53* and *BRCA1* target sequences and inserted the oligonucleotides into the pMXs-mU6-puro plasmid, as shown in our previous report [53]. The shRNA sequences of *TP53*, *BRCA1* and the negative control (sh*GFP*) were as follows: sh*TP53* 5′-GGA TTT CAT TTC TTG TGT ATG GTT CAA GAG ATC ATA TAC AAG AGA TGA AAT CCT TTT T-3′ and 5′-CGA AAA AGG ATT TCA TCT CTT GTA TAT GAT CTC TTG AAC CAT ACA CAA GAA ATG AAA TCC-3′, sh*BRCA1* 5′- GAA AGA AGT GGA TTT GTC TGT TTC AAG AGA GCA GAT AAA TCC ATT TCT TTC TTT TT-3′ and 5′-CGA AAA AGA AAG AAA TGG ATT TAT CTG CTC TCT TGA AAC AGA TAA ACC CAT TCC TTT C-3′, and sh*GFP* 5ʹ-GTG CTA TTG GAG TTG ATA GTC TTC AAG AGA GAT TAT CAA TTC CAA TAG TAC CTT TTT-3ʹ and 5ʹ-CGA AAA AGG TAC TAT TGG AAT TGA TAA TCT CTC TTG AAG ACT ATC AAC TCC AAT AGC AC-3ʹ. Underlined letters indicate loop structure sequences. sh*TP53*-, sh*BRCA1*- and sh*GFP*-expressing MCF-7 cells were generated using a retroviral system with plat-A cells as previously reported [53]. In brief, each pMXs-mU6-puro plasmid was transfected into plat-A cells using FuGENE 6 (Promega, Tokyo, Japan). The supernatant from each virus-containing culture was collected 2 days later and concentrated with 4× PEG-it solution [32% (w/v) PEG-6000, 400 mM NaCl, and 40 mM HEPES, pH 7.4]. MCF-7 cells were incubated with virus particles for 24 h, followed by selection with 2 μg/mL puromycin for 5–7 days.

### Transfection of siRNAs

For the MDM2, CHFR or RNF146 transient KD, we introduced two Silencer Select Pre-Designed siRNAs against *MDM2* (si*MDM2*: s224037 and s8628), *CHFR* (si*CHFR*: s31392 and s31393) or *RNF146* (si*RNF146*: s37822 and s37823) (Thermo) into MCF-7 cells using Lipofectamine RNAiMAX Transfection Reagent (Thermo), respectively. Silencer Select Negative Control #1 siRNA (Thermo) was used as a negative control. Transfection of siRNAs was performed according to the manufacturer’s protocol. Cells were treated with 10 μM nutlin-3a at 48 h after transfection of si*MDM2* or 60 h after transfection of si*CHFR* or si*RNF146*. After 24 h, treated cells were collected and analyzed.

### Immunoblotting analysis

Immunoblotting was performed as previously reported [27]. Briefly, collected cells were lysed with SDS sample buffer [50 mM Tris-HCl pH 6.8, 2% SDS, 3 M urea, 6% glycerol], boiled for 5 min and sonicated. Next, lysates were subjected to SDS-PAGE on 10% poly-acrylamide gels and transferred to nitrocellulose membranes. The membranes were blocked with blocking solution [2.5% skim milk and 0.25% BSA in TTBS (50 mM Tris, pH 7.4, 150 mM NaCl, 0.1% Tween 20)] for 60 min at room temperature and then probed with the appropriate primary antibody overnight at 4°C: PARP1 (9532; Cell Signaling, Beverly, MA, USA), p53 (clone DO-1, OP43L; Calbiochem San Diego, CA, USA), CASP7 (9492; Cell Signaling), glyceraldehyde-3-phosphate dehydrogenase (GAPDH) (sc-32233; Santa Cruz Biotechnology, San Francisco, CA, USA), or pADPr (clone 10H, sc-56198; Santa Cruz Biotechnology). The membranes were then incubated with the appropriate secondary antibody for 60 min at room temperature: horseradish peroxidase-conjugated F (ab’)2 fragment of goat anti-mouse IgG (115-036-062) or anti-rabbit IgG (111-036-045) (Jackson Immuno Research, West Grove, PA, USA). Chemiluminescence was performed with ImmunoStar LD Reagent (Wako). The antibody-bound proteins were visualized with an LAS3000 Image Analyzer (Fujifilm, Tokyo, Japan), and data were analyzed using Multigauge software (GE Healthcare, Madison, WI, USA). GAPDH was used as a loading control.

### RNA extraction and quantitative RT-PCR (qRT-PCR)

Total RNA was extracted from cells using ISOGEN II (Nippon Gene, Tokyo, Japan) according to the manufacturer’s protocol. Purified RNA (0.5 μg) was subjected to reverse transcription with the ReverTra Ace qPCR RT Master Mix with gDNA Remover (TOYOBO, Osaka, Japan). qRT-PCR was performed using a CFX Connect™ RT-PCR System (Bio-Rad, CA, USA) with THUNDERBIRD SYBR qPCR Mix (TOYOBO). The quantitative PCR data were processed with a standard curve method. β-Actin was used for normalization. The primer sequences are shown in Table 1.

**Table 1:** Primers used in the present study

Genes	Forward (5′ to 3′)	Reverse (5′ to 3′)
*MDM2*	GCTGGGAACCTCTTGATTGTG	ATCCACCCATAAAGCGCAAC
*CHFR*	TTCTGTGGAGCTTTACCCTCTG	GATAAACTTGCCCTTCTCCCTTG
*RNF146*	CAAACAGGAAAGCGAACGAG	TTCTGGTGACAACAAGGTTGG
*BRCA1*	AACCAGGAGTGGAAAGGTCATC	GTTTCCGTCAAATCGTGTGG
*β-ACTIN*	TGGGACGACATGGAGAAAATC	ATAGCACAGCCTGGATAGCAAC

### Measurement of cell viability

The number of viable cells was determined using the Cell Counting Kit-8 (DOJINDO, Kumamoto, Japan) according to the manufacturer’s protocol. Briefly, cells were plated at a density of 5000 cells/well in 96-well plates. After 1–2 h, cells were treated with 10 μM nutlin-3a for 12 h and then 1, 5, 10, 20, 40 or 60 μM cisplatin was added. After 24 h, 10 μL Cell Counting Kit reagent was added to wells, and cells were incubated at 37°C for 3 h. Absorbance was measured at 450 nm using an ARVO MX/Light Wallac 1420 Multilabel/Luminescence Counter (PerkinElmer, Waltham, MA, USA). Cell viability was calculated according to the manufacturer’s protocol for the Cell Counting Kit-8.

### Statistical analysis

Data were statistically evaluated by the Student *t* test, the Tukey’s test or Dunnett’s test using BellCurve for Excel software (Social Survey Research Information Co., Ltd, Tokyo, Japan). Data are presented as mean ± standard error of the mean (SEM), and *p* < 0.05 was considered significant.
